# Targeting Pancreatic Cancer Cell Plasticity: The Latest in Therapeutics

**DOI:** 10.3390/cancers10010014

**Published:** 2018-01-10

**Authors:** Jacob M. Smigiel, Neetha Parameswaran, Mark W. Jackson

**Affiliations:** 1 Department of Pathology, Case Western Reserve University, Cleveland, OH 44106, USA; jxs1094@case.edu (J.M.S.); nxp169@case.edu (N.P.); 2 Case Comprehensive Cancer Center, Case Western Reserve University, Cleveland, OH 44106, USA

**Keywords:** cancer stem cells, cell plasticity, epithelial–mesenchymal transition, tumor microenvironment, oncogenes, therapeutics

## Abstract

Mortality remains alarmingly high for patients diagnosed with pancreatic ductal adenocarcinoma (PDAC), with 93% succumbing to the disease within five years. The vast majority of PDAC cases are driven by activating mutations in the proto-oncogene KRAS, which results in constitutive proliferation and survival signaling. As efforts to target RAS and its downstream effectors continue, parallel research aimed at identifying novel targets is also needed in order to improve therapeutic options and efficacy. Recent studies demonstrate that self-renewing cancer stem cells (CSCs) contribute to metastatic dissemination and therapy failure, the causes of mortality from PDAC. Here, we discuss current challenges in PDAC therapeutics, highlight the contribution of mesenchymal/CSC plasticity to PDAC pathogenesis, and propose that targeting the drivers of plasticity will prove beneficial. Increasingly, intrinsic oncogenic and extrinsic pro-growth/survival signaling emanating from the tumor microenvironment (TME) are being implicated in the de novo generation of CSC and regulation of tumor cell plasticity. An improved understanding of key regulators of PDAC plasticity is providing new potential avenues for targeting the properties associated with CSC (including enhanced invasion and migration, metastatic outgrowth, and resistance to therapy). Finally, we describe the growing field of therapeutics directed at cancer stem cells and cancer cell plasticity in order to improve the lives of patients with PDAC.

## 1. Introduction: The Alarming Context of Pancreatic Cancer

Malignancies of the pancreas can be subdivided into two main categories, those arising from exocrine cells, which produce digestive enzymes, and those from endocrine cells, which produce and release hormones such as insulin and glucagon [1]. Upwards of 95% of the new cases of pancreatic cancers are tumors originating from the exocrine gland and are referred to as pancreatic ductal adenocarcinomas (PDACs). Unfortunately, the statistics for patients with PDAC are grim; nearly as many patients die from PDAC as are diagnosed each year, and 93% of patients succumb to the disease within 5 years of their first diagnosis (20% within their first year). There are a number of reasons associated with the poor prognosis associated with PDAC. Many patients present in the clinic with widespread metastatic disease, as no or minimal symptoms of PDAC are evident until the disease has progressed to later stages. Surgical resection is feasible only in patients with rare localized disease, leaving most to receive generalized chemotherapy [2], which improves median survival by a mere 6 months due to acquired resistance to therapy [3,4]. Strikingly, the incidence and rates of death from PDAC have begun to move upward in the past 2–3 years, with the disease now projected to be the second leading cause of cancer-related death [5,6]. Together, these observations underscore the need to identify early detection methods and the contributors to aggressive features of PDAC so that new therapies can be developed to combat this devastating disease.

PDAC develops from normal pancreatic epithelium, which transitions first through a non-malignant, neoplastic state referred to as pancreatic intraepithelial neoplasm (PanIN) before culminating in a fully transformed state, and this transformation relies heavily on the early mutation of the oncogene KRAS, with ~90% of PDACs possessing activating RAS mutations [7]. Hyperactive RAS signaling not only drives tumor formation and maintenance but also contributes to metastatic dissemination and therapy failure [8,9,10]. Despite its central importance in PDAC development and progression, attempts to target mutant KRAS have been largely unsuccessful. Furthermore, the microenvironment surrounding PDAC, comprised of numerous cell types (endothelial cells, pancreatic stellate cells, fibroblasts, neurons, and immune cells), contributes to the aggressiveness of the disease [11,12,13]. Here, we will discuss the aggressive nature of PDAC and the challenges we currently face in treating the disease.

**Figure 1:**
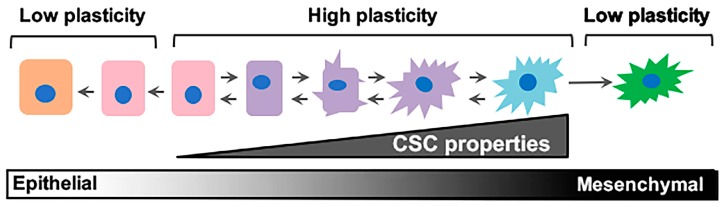
Epithelial/non-stem cell to mesenchymal/cancer stem cell plasticity. Epithelial/non-cancer stem cells retaining cell plasticity respond to environmental or intrinsic cues by fluidly transitioning through intermediary stages until reaching a mesenchymal/cancer stem cell state. These intermediary states hold immense plasticity and are the roots behind metastatic dissemination and therapeutic failure. CSC: cancer stem cell.

As in many other cancer types, research is uncovering how PDAC cells adapt to varying stimuli (hypoxia, chemotherapy, immune cell infiltration, etc.) by changing cell state. PDAC cells that have an ability to undergo reprograming as the tumor microenvironment (TME) changes are said to have cellular plasticity. The most prominent example of cellular plasticity occurs when an epithelial cancer cell transitions into a migratory, invasive, mesenchymal cell, in a process called epithelial–mesenchymal transition (EMT). This transition involves passing through a series of intermediate states (Figure 1), with some cells expressing both epithelial and mesenchymal proteins. Studies linking EMT with the acquisition of cancer stem cell (CSC) properties provide important context for understanding the relationship between epithelial/non-CSCs and mesenchymal/CSCs (Mes/CSCs), as well as the hybrids between these states. We refer to plasticity throughout this review as the cell’s ability to fluidly move between these cell states. While we focus mainly on epithelial–mesenchymal (E–M)/CSC plasticity, we acknowledge that cells along this spectrum may utilize metabolic processes differently, engage immune cells differently, and respond differently to any number of environmental changes [14,15,16].

Obviously, not all cells are able to respond as fluidly to a changing environment (low plasticity), but those cells that do appear to be an important driving force behind metastatic dissemination and therapy failure. As new insights into the dynamic changes in PDAC cell states and the signaling pathways that contribute to plasticity are defined, new opportunities for targeting the deadly nature of PDAC will emerge. Here, we discuss how tumor cell intrinsic and extrinsic signals from the tumor microenvironment contribute to E–M/CSC plasticity and delve into how identifying novel factors that contribute to E–M/CSC plasticity can lead to the next generation of therapeutics aimed at extending patient survival.

## 2. PDAC Therapeutics: Challenges and Opportunities

In the last 40 years, survival rates for patients with PDAC have remained unchanged, while patients with nearly every other cancer type have seen remarkable increases in survival. This is due, in part, to a lack of early diagnostic measures and the absence of efficient therapeutic options following diagnosis. Only 25% of patients have localized tumors eligible for surgery; the vast majority present with advanced, metastatic disease that cannot be treated with surgery, leaving these patients to receive radiation or chemotherapy. To date, the mainstay therapy for PDAC remains single-agent treatment with gemcitabine, a deoxycytidine analog. Recently, FOLFIRINOX, the combination of 5-fluorouracil (5-FU), leucovorin, irinotecan, and oxaliplatin, has proven beneficial, despite complicated side effects of neutropenia and thrombocytopenia [17]. In the last decade, a better understanding of the genetics and molecular machinery that drive PDAC has resulted in the approval of the epidermal growth factor receptor (EGFR) tyrosine kinase inhibitor erlotinib as the first, and thus far only, targeted therapy [18]. By combining erlotinib with gemcitabine in patients with advanced metastatic PDAC, increases in both one-year (23% vs. 17%) and median (6.24 months vs. 5.9 months) survival have been achieved [19]. While this is a modest improvement in survival when compared to gemcitabine alone, the combination marked a significant step towards expanding the therapy choices for patients. Interestingly, recent use of nanopartical albumin-bound paclitaxel (nab-P) in conjunction with gemcitibine has shown a marked improvement over gemcitibine alone. Approved in 2013 by the FDA, nab-P/gemcitibine regimens have showed promise in treatment of late-stage metastatic pancreatic cancer [20,21]. This drug regimen is becoming standard of care for both early- and late-stage patients, with patient overall survival increasing by 2 months (from 6.7 with gemcitabine alone, to 8.5 with combination nab-P/gemcitibine) [22].

Not surprisingly given the prevalence of RAS mutations in PDAC, early drug development efforts focused on targeting mutant KRAS and downstream RAS effectors, including Rapidly Accelerated Fibrosarcoma (RAF)/Mitogen Activated Protein Kinase (MEK)/Extracellular Signal-Regulated Kinases (ERK) and Phosphatidylinositol-4,5-bisphosphate 3-kinase (PI3K)/Protein Kinase B (AKT) signaling responsible for driving cancer cell proliferation and survival [23]. However, targeting mutant RAS has been a struggle for a number of reasons. Early farnesyltransferase inhibitors (FTIs) designed to prevent the farneyslation and consequent membrane localization of RAS were ineffective in the clinic because of compensatory geranylation, which also facilitates RAS membrane localization [24]. Following the failure of FTIs, mutant RAS was deemed “undruggable” and there was a paucity of research aimed at targeting RAS until 2013, when the National Cancer Institute established a RAS Initiative to support new approaches aimed at inhibiting the function of mutant RAS.

One strategy that is proving effective is to block the interactions between RAS and binding partners responsible for transmitting signals. A notable clinical trial for advanced pancreatic cancers is assessing rigosertib, which blocks RAS from associating with the RAS-binding domain of RAF, thereby preventing RAF/MEK/ERK signaling [25]. Additional promising drugs include deltarasin [26], which blocks RAS-Phosphodiesterase [delta] (RAS-PDEδ) interactions, and small molecules that prevent Son of Sevenless (SOS1), a RAS-guanine exchange factor (GEF), from binding to RAS, keeping it in an inactive Guanosine Diphosphate (GDP)-bound form [27]. Antisense oligonucleotides that directly target RAS (e.g., ISIS 2503) performed well in phase I clinical trials for metastatic PDAC [28,29]. It remains to be seen how these new approaches to target mutant RAS will fare in the rigors of clinical testing. Other approaches aim to undermine the effectors activated downstream of mutant RAS, with numerous clinical trials testing MEK inhibitors (MEK162, trametinib, pimasertib), ERK inhibitors (BVD-523) and PI3K inhibitors (BKM120, BEZ235) in PDAC [30]. Thus far however, targeting these effectors individually has been fraught with problems of toxicity and the rapid emergence of resistance due to the feedback activation of the compensatory signaling. Feedback activation loops involving Signal Transducer and Activator of Transcription 3 (STAT3) have also been reported as a result of MEK inhibition [31,32,33]. Concurrent inhibition of these signaling pathways at multiple levels may overcome the problem of feedback activation.

While new drugs continue to be developed and improved upon, one of the major hurdles to be addressed in pancreatic cancer therapeutics is the inaccessibility of the tumors to the administered drugs. A fundamental characteristic of PDAC is the presence of desmoplasia, an alteration and remodeling of the surrounding tumor stroma [34]. This desmoplasia typically manifests itself through an over-production of extra cellular matrix proteins (e.g., collagen types I, III, and IV, fibronectin, laminin, and hyaluronan) as well as proliferation of myo-fibroblast like cells [35,36]. This results in extensive fibrosis surrounding the tumor comprising both cellular and non-cellular components. The combination of desmoplasia and fibrosis makes PDAC dense and avascular at the core, protecting tumor cells from exposure to systemic therapies. Additionally, metastatic lesions of PDAC, including those in the liver, lung, and the peritoneal cavity, have comparable levels of desmoplasia and fibrosis when compared to primary PDAC [37]. Moreover, the presence of increasing desmoplasia and fibrosis correlates with poor patient survival, in part because of the poor perfusion and inaccessibility to systemic therapies, but also because of perturbed cytokine network surrounding the tumor [38,39].

Approaches to overcome this hurdle by targeting the desmoplasia and fibrosis involve the use of adhesion kinase inhibitors that disrupt tumor cell–stroma interactions. In preclinical testing, Focal Adhesion Kinas (FAK) inhibitors significantly reduced desmoplasia and increased immune surveillance [40], resulting in a clinical trial combining the FAK inhibitor GSK2256098 with the MEK inhibitor trametinib. Inhibition of the sonic hedgehog pathway to reduce tumor stroma and increase tumor vasculature, allowing increased access of drugs to tumor cells, showed great promise in early clinical studies [41]. However, later trials were abandoned due to increased adverse events associated with highly vascular tumors [42].

## 3. The Elusive Problem Child: Epithelial–Mesenchymal/Cancer Stem Cell (E–M/CSC) Plasticity

Metastatic dissemination and therapeutic resistance leading to tumor recurrence are the main reasons behind mortality associated with PDAC. The observation that most patients present with metastatic disease at diagnosis, coupled with studies demonstrating metastatic dissemination can precede clinically evident malignancy, emphasizes the importance of understanding pathways that contribute the metastatic cascade in PDAC [43,44]. Metastasis of epithelial cancer cells is a dynamic process which relies heavily on a cell’s ability to transition between epithelial and mesenchymal states. During epithelial-to-mesenchymal transition (EMT), tight cell–cell junction proteins such as ZO-1, occludins, and E-cadherins become repressed by the key transcriptional regulators Zinc Finger E-Box Binding Homeobox 1 (ZEB1), Zinc Finger Protein SNAI1 (SNAI1), and Twist Family BHLH Transcription Factor 1 (TWIST1) [45,46,47]. The loss of tight cell–cell junctions along with the upregulation of extracellular matrix remodeling proteinases allows the cells to invade surrounding tissues and enter into the lymphatics or bloodstream, promoting widespread dissemination [48,49,50,51].

Circulating tumor cells (CTCs) have elevated levels of the key transcription factors ZEB1, SNAI1, and TWIST and their downstream targets [52]. In patients with breast cancer, the presence and abundance of CTCs expressing mesenchymal markers can be used to track a patient’s response to therapy. In patients that respond to therapy, mesenchymal CTC numbers decrease, while patients with progressive disease have increased numbers of mesenchymal CTCs. Moreover, the re-emergence of mesenchymal CTCs in patients that initially responded to therapy is accompanied by tumor recurrence. Consistent with these observations, key evidence from seminal work by Mani et al. and Morel et al. now links EMT with the acquisition of cancer stem cell (CSC) properties [53,54]. By definition, CSCs can recapitulate the heterogeneity of the primary tumor from which they were isolated following orthotopic transplantation into mice. As the progeny of CSCs differentiate, they lose their ability to generate tumors, despite their identical genetic landscape. CSCs have been isolated from nearly every type of human malignancy using a limited (albeit non-overlapping) set of markers. Importantly, CSCs display a decreased sensitivity to chemo- and radiation-therapy [55,56,57,58,59], and display enhanced invasiveness when compared to cells lacking CSC markers. Likewise, a sub-set of CTCs responsible for initiating metastasis (termed the metastasis-initiating cells) has been identified as expressing the CSC marker Cluster of Differentiation 44 (CD44).

In PDAC, the ability to survive therapy, engage in self-renewal, and initiate tumor formation with limited cell numbers are hallmarks of the stem cell state [60,61,62]. As in breast cancer, PDAC CTCs possess elevated CSC markers (CD44, Cluster of Differentiation (CD133), and Aldehyde Dehydrogenase 1 (ALDH1)) together with elevated mesenchymal markers, making the case that mesenchymal/CSCs contribute to metastatic dissemination and therapeutic resistance. The significance of a panel of CSC markers (CD133, CXCR4, CD44, CD24) were originally identified by Li et al. [61]. Using patient-derived xenograft models, isolated CD44+/CD24+/ESA+ cells, representing between 0.2% and 0.8% of the PDAC population, were shown to have nearly 100-fold greater tumor-initiating capacity when compared to cells lacking these markers [61]. However, other key studies by Hermann et al. have identified CD133+/CXCR4+ PDAC cells as possessing an enhanced metastatic capacity in addition to their tumor-initiating capacity when compared to CD133−/CXCR4– populations [63].

**Figure 2:**
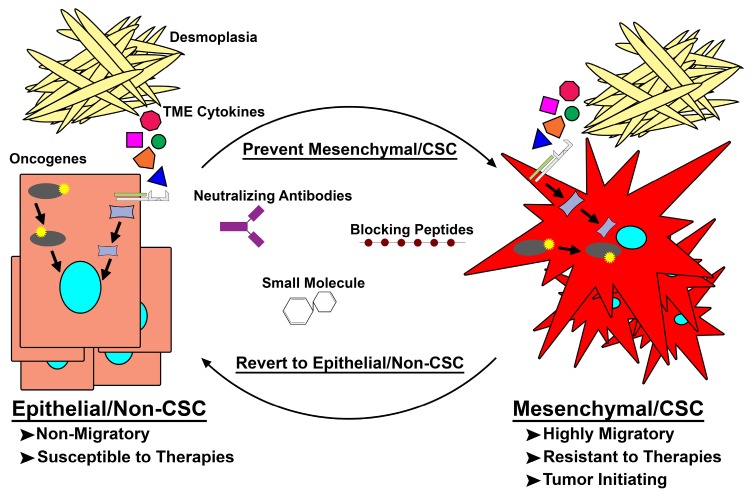
Targeting de novo mesenchymal/cancer stem cell (Mes/CSC) plasticity. Epithelial/non-cancer stem cells acquire mutations within oncogenes or are exposed to extrinsic factors capable of driving them to a mesenchymal/CSC (Mes/CSC) state. This Mes/CSC plasticity imparts increased migration and metastases, tumor initiation, and importantly therapeutic resistance. In order to combat this cell plasticity, one can utilize neutralizing antibodies targeting extrinsic drivers (i.e., Oncostatin M (OSM), Transforming Growth Factor-β (TGF-β), Tumor Necrosis Factor-α (TNF-α)), and blocking peptides or small molecules targeted toward intrinsic drivers (i.e., Family with Sequence Similarity 83 Member A (FAM83A), KRAS, PI3K) of cell plasticity to prevent Mes/CSC induction or revert the Mes/CSC state into a more drug sensitive epithelial/non-CSC state. TME: tumor microenvironment.

Early hypotheses purported that CSCs arose from transformed tissue stem cells [64]. Conventional nomenclature has dubbed these cells innate or pre-existing CSCs [65,66,67,68,69]. It has long been thought that these pre-existing CSCs, and the plasticity associated with them, are responsible for tumor heterogeneity, drug resistance, and metastatic outgrowth. While targeting pre-existing CSCs may prove beneficial to patients, a larger challenge remains. Recent studies confirm that transition towards a more mesenchymal/CSC state can occur spontaneously or in response to TME changes [70,71]. Our own work, along with others, has shown the generation of a spontaneous Mesenchymal/CSC population possessing increased CD44, ZEB1, and SNAI1 expression and a reduction in CD24 and E-cadherin during the transformation of human mammary epithelial cells (HMECs) [72,73,74]. However, these populations lack the ability to revert to a more epithelial/non-CSC state, suggesting a lack of cellular plasticity. More recently, a number of cell-intrinsic and external stimuli (i.e., oncogenes, cytotoxic therapies, and TME cytokines) were shown to induce remarkable cellular plasticity within epithelial/non-CSCs, driving these epithelial/non-CSC cells through a de novo transition to a Mes/CSC state [8,70,74,75,76] (Figure 2). This de novo transition to a Mes/CSC cell state describes another possible outcome to the CSC theory, wherein a sub-population of epithelial cells which do not possess invasive or stem-like properties respond to external or internal signals by transitioning to a CSC state. This phenomenon of a de novo transition is unique from previously discovered inherent or “pre-existing” CSC. Moreover, the ability of non-CSCs to acquire CSC properties complicates efforts to target CSCs in PDACs, as cells capable of being induced into a drug-tolerant state, escaping therapeutic regimens and promoting heavier metastatic burden, will likely require distinct therapies (Figure 2). Understanding the pathways regulating CSC plasticity will be an important next step in combating metastatic and therapy-resistant tumors such as PDAC [63,77].

## 4. Intrinsic and Extrinsic Drivers of E-M/CSC Plasticity 

E–M/CSC plasticity allows cells to switch between cell states (epithelial versus mesenchymal, differentiated versus less-differentiated, stem-like cell states). These transitions are often manifested by multiple factors, including but not limited to extrinsic secreted factors in the tumor microenvironment, metabolic stress such as hypoxia, or tumor-intrinsic drivers. Krebs et al. demonstrated a significant reduction in E–M/CSCs following ZEB1 knock-out resulted in the suppression of metastatic outgrowth in the murine KRAS^G12D^/p53^R172H^KPC mouse model [70]. Furthermore, oncogenic signaling has been shown to be a tumor-intrinsic driver of E-M/CSC plasticity in PDAC. For example, several newly identified oncogenic and pro-growth/survival signaling pathways feed into the generation and maintenance of CSC. We and others have shown that the oncogenic Family with Sequence Similarity 83, Member A (FAM83) family of proteins contribute to drug resistance in addition to driving transformation. As reported in breast cancer [78], high expression of FAM83B in PDAC may be an important prognostic marker as it positively correlates with shorter survival and advanced clinical disease [79]. Both human and murine pancreatic cancers also significantly overexpress the smallest member of the FAM83 protein family, FAM83A (originally named BJ-TSA-9) which has been shown to drive tumor cell survival via the MEK/ERK signaling pathway [80,81]. FAM83A overexpression was reported by Chen et al. to activate Wnt/β-catenin signaling and induce the emergence of CSC properties including drug resistance in pancreatic cancer cells [82]. Importantly, FAM83A was identified as one of three markers that can be used to identify CTCs in the peripheral blood of patients with breast and lung cancer [83,84]. FAM83A RNA could even be detected in early-stage (stage I and II) patients, suggesting that it may be valuable as an early detection biomarker for PDAC as well [84]. In addition, the detection of FAM83A in peripheral blood correlated with poor response to therapy and shorter survival times, suggesting that it may also be used as a predictive factor. Likewise, a multi-omics study identified a FAM83 gene family signature as a marker of poor prognosis in many human cancers including pancreatic cancers [85].

In addition to cancer cell-intrinsic genetic alterations, the TME can influence tumor development and progression, although little remains known about the intricacies of the stromal-tumor cell crosstalk during PDAC development [86]. In experimental models of PDAC progression, tumor cells respond to TME cytokines by acquiring stem cell properties that can have a profound impact on tumor heterogeneity. Cells harboring CSC properties are afforded an enhanced ability to survive therapy and respond to the drastically-changing TME following therapy, thereby repopulating the tumor at the primary or metastatic sites. Transforming Growth Factor-β (TGF-β) and Interleukin 6 (IL-6) have been shown to induce mesenchymal/CSC properties in certain PDAC model systems [87]. Our studies implicate the TME cytokine oncostatin-M (OSM) as a potent inducer of mesenchymal/CSC properties in PDAC cells, including tumor-initiating capacity, metastasis, and resistance to gemcitabine (Figure 1) [76]. OSM is elevated in the serum of PDAC patients and serves to predict poorer response to therapy [88], suggesting its promise as a therapeutic target. Targeting TME cytokines or their receptors with neutralizing antibodies would potentially prevent or reverse the acquisition of CSC properties underlying metastasis and therapy failure (Figure 1). In the case of OSM, it is an inflammatory cytokine produced by tumor stromal cells, infiltrating immune cells, or the cancer cells themselves; we are now working to capitalize on the fact that OSM has already been targeted for treatment of other inflammatory diseases [89,90], both in mice and humans, without any serious toxicity issues [91].

Aberrant TME resulting from increased desmoplasia has been linked to aberrant metabolism and chemotherapy resistance in PDAC. Tumor cells make intrinsic adjustments to survive in the nutrient- and oxygen-deprived niche by altering glucose metabolism, utilizing glutamine instead of depending on glucose and upregulating scavenger pathways of macroautophagy [92]. Recently, Shukla et al. demonstrated that Hypoxia Inducible Factor-1α (HIF-1α) stabilization and signaling induces anabolic glucose metabolism and increased pyrimidine biosynthesis, thereby imparting gemcitabine resistance to pancreatic cancers [93]. Since hypoxia is known to induce stem cell transcription factors [94], Nanog and Oct4, and pancreatic CSC markers CD133 [63], and CXCR4 [95], one may speculate that the resistance is accompanied by the acquisition of a CSC state. Hypoxia induces expression of proangiogenic factor Vascular Endothelial Growth Factor (VEGF), Matrix Metallopeptidase-3 (MMP3), and inflammatory cytokine IL-6, all reported to induce invasiveness of pancreatic cancers [94,96,97]. Inhibitors of HIF-1α, VEGF, and MMP3 are all in consideration for future therapeutics targeting pancreatic CSCs. Activated pancreatic stellate cells (PSCs) are stromal cells in the pancreatic tumor microenvironment that are known to induce mesenchymal/CSC-properties in neighboring epithelial cancer cells [98]. PSCs activated in response to inflammatory cytokines, growth factors, or oxidative or hypoxic stress provide an optimal niche for supporting pancreatic CSCs [99], suggesting that PSC-targeting therapies combined with chemotherapy may be effective in minimizing therapy-failure. In addition to the critical tumor cell–stromal interactions, the tumor cell–immune cell interactions also contribute to cancer cell plasticity. B cell-activating factor (BAFF), a cytokine secreted by B lymphocytes infiltrating pancreatic cancers, induces EMT, resulting in enhanced motility and invasiveness of pancreatic epithelial cells [100].

## 5. Latest in Mes/CSC Therapeutics and the Future of Targeting Mes/CSC Plasticity

While the concept of cellular plasticity is fairly new and targeting drivers of this process holds significant promise, further studies to identify and target drivers of Mes/CSC plasticity are still needed. However, since therapeutic resistance has been heavily contributed to the CSC phenotype the attempt at targeting pathways which pre-existing or inherent CSC are dependent on have been an avenue of study for some time [101,102,103]. Attempts at re-sensitizing CSC to current cytotoxic therapies are also underway and many studies have shown novel CSC inhibitors can increase the efficacy of current cytotoxic therapies, however these therapies may only kill pre-existing CSCs and not effectively target populations of cells undergoing Mes/CSC plasticity [104,105,106,107,108,109]. These targets include but are not limited to monoclonal Antibody (mAB) targeting CSC-specific surface markers, STAT3 signaling, and oncogenic Pi3K/Mammalian Target of Rapamyacin (mTOR) signaling. It is evident that induction and maintenance of the CSC population within PDAC relies heavily on signaling activated by oncogenes, cytokines, and growth factors, which likely work through redundant pathways. Therefore, targeting intracellular kinases may hinder CSC generation and/or maintenance (Figure 2). The promiscuous, multi-kinase inhibitor sorafenib, for example, can target PDAC CSCs, suppressing colony outgrowth, tumorsphere formation, ALDH1 activity, and tumor-initiating capacity [110]. Additionally, targeting hyperactive MAPK or Wnt/β-catenin signaling responsible for inducing and maintaining CSC properties in combination with standard chemotherapy holds great promise in eliminating recurrence. Suzuki et al. demonstrated the c-Jun N-Terminal Kinases (JNK) pathway as a crucial signaling axis in pancreatic CSC population, and targeting JNK signaling increased PDAC CSC sensitivity to 5-FU or gemcitabine [111]. Likewise, targeting mTOR, a key regulator of the stem-like phenotypes in PDAC, in combination with c-MET signaling showed a striking reduction in the viability of a CD133+ CSC population [112,113]. Resveratrol, a polyphenolic phytochemical, improves the anti-tumor activity of gemcitabine by reducing ZEB1, Slug, and Snail expression as well as Nanog and Oct4 expression and that of drug-resistant ATP-Binding Cassette (ABC) transporter genes, in addition to pathways governing the self-renewal of CSCs [114]. Another natural compound, genistein, inhibits Notch signaling, thereby reducing the self-renewal of pancreatic CSCs [115]. Direct targeting of newly implicated growth promoting oncogenes, such as FAM83A, that have tumor-exclusive expression offers a significantly better therapeutic window and achieves inhibition of multiple oncogenic signaling.

While novel oncogenic signaling is promising, other targets utilizing small molecules inhibitors of molecular targets other than kinases have shown promise. CD133+ CD44+ pancreatic CSCs were shown to be less reliant on glycolysis and sensitive to inhibitors of oxidative phosphorylation such as oligomycin that reduced spherogenic potential and regressed tumors in mice [116]. Whereas cytotoxic gemcitabine induces apoptosis mainly in non-CSC, CD133– PDAC cells, salinomycin, an antibiotic isolated from *Streptomyces albus*, selectively targets tumorsphere initiation and growth in CD133+ PDAC CSCs. When combined, salinomycin and gemcitabine drastically reduced the human pancreatic xenograft model’s outgrowth compared to either agent alone [117]. Interestingly, Zhang et al. showed the ability for aspirin to target PDAC CSCs by reducing self-renewal and inducing apoptosis in vivo [118]. A recently discovered metabolic drug, metformin, which was originally employed to combat diabetes by acting on the liver, has been shown to interfere with insulin receptor signaling within pancreatic cancer [119,120]. Metformin has been shown to inhibit pancreatic cell invasion and tumorsphere formation, but more importantly prevents the progression of PDAC in genetically-engineered mice by targeting the CSC population [120,121]. Metabolic reprogramming and dependency has become a focal point of targeting cancer stem cells recently, as Lonardo et al. and Sancho et al. demonstrated metformin’s ability to target PDAC CSCs. Their work demonstrated PDAC CSC dependency on mitochondrial oxidative phosphorylation (OXPHOS), and inhibition by metformin caused rapid apoptosis in CSCs while non-CSC populations merely underwent cell cycle arrest. These findings suggest that targeting the dependency of metabolic reprogramming of cells undergoing cell plasticity may prove beneficial [122,123]. Similarly, targeting CD44 or inhibiting ABC transporters using verapamil was correlated with decreased CSC frequency and re-sensitization to gemcitabine [77].

Others have harnessed the immune system in PDAC. For instance, Chimeric Antigen Receptor (CAR) T cells engineered to target a native protein mesothelin overexpressed on pancreatic cancer cells show significant anti-tumor activity independent of chemotherapy or radiation [124]. Harnessing T-cell influx has shown promise with the use of vaccines in conjunction with current PDAC therapies. GVAX, a vaccine generated from two pancreatic cell lines engineered to secret granulocyte-macrophage colony-stimulating factor, increases immune efflux and prolongs disease-free survival [125,126]. Similarly, use of algenpantucel-L, an irradiated allogeneic pancreatic cancer cell vaccine that expresses murine α-1,3-galactosyltransferase, induces antibody-dependent cell cytotoxicity (ADCC) and immune response against human pancreatic cancer cells. In a phase II trial, overall and disease-free survival was improved for those receiving combined algenpantucel-L and cytotoxic therapy as opposed to cytotoxic therapy alone [127]. Immune checkpoint inhibition to boost anti-tumor T cell responses are also being examined for efficacy in PDAC patients with clinical trials ongoing for anti-Cytotoxic T-Lymphocte-Associated Protein 4 (CTLA-4) (ipilimumab, tremilimumab), anti-PD1 (nivolumab, pembrolizumab), and anti-PDL1 (BMS-936559) blocking antibodies. However, immune activation is not always beneficial, as the presence of some immune cytokines can drive Mes/CSC plasticity. For cytokines that drive CSC properties (TNF α, TGF-β, IL-6, and OSM), we propose a more “outside-in” approach to targeting the disease by using neutralizing antibodies targeting the cytokine or the receptor in order to prevent CSC generation or maintenance, to minimize the outgrowth of disseminated cells or prevent therapy failure.

## 6. Conclusions

While a lot of effort has been put into targeting CSC populations, these novel therapies may only be effective at killing pre-existing CSC and not cells undergoing cellular plasticity. A better understanding of how epithelial cancer cells are reprogrammed into mesenchymal/CSC is needed, as more avenues of targeting their generation and maintenance must be pursued. It is evident however, that targeting Mes/CSCs poses new challenges with respect to the timing and specific target needed to alter this cellular plasticity. There is also an urgent need to improve on experimental model systems to better represent the PDAC tumor microenvironment (co-culture or co-implantation of cancer cells with stromal cells and immune cells) to identify drivers of Mes/CSC plasticity. Recently, dendritic cells primed with pancreatic CSC lysates were shown to have increased anti-tumor activity, which may serve as a feasible strategy for targeting these difficult to kill cells. The future may envision chimeric antigen receptor therapy targeting the circulating and intra-tumoral Mes/CSC populations. It will be of utmost importance to identify targets of Mes/CSC plasticity exclusive to cancer tissues so as to increase the therapeutic index and maintain the patient’s quality of life.
